# What Is the Most Appropriate Induction Regimen for the Treatment of HIV-Associated Cryptococcal Meningitis When the Recommended Regimen Is Not Available? Evidence From a Network Meta-Analysis

**DOI:** 10.3389/fphar.2020.00963

**Published:** 2020-06-30

**Authors:** Yao Li, Xiaojie Huang, Yuanyuan Qin, Hao Wu, Xiaofeng Yan, Yaokai Chen

**Affiliations:** ^1^ Division of Infectious Diseases, Chongqing Public Health Medical Center, Chongqing, China; ^2^ Center for Infectious Diseases, Beijing Youan Hospital, Capital Medical University, Beijing, China

**Keywords:** HIV, cryptococcal meningitis, antifungal regimen, induction treatment, network meta-analysis

## Abstract

**Aims:**

Our object was to find the most appropriate, most effective, and most readily available of four induction regimens for HIV-associated cryptococcal meningitis (CM) (Regimen A: 1 week of AmB plus 5-FC followed by 1 week of fluconazole, Regimen B: 1 week of AmB plus fluconazole followed by 1 week of fluconazole, Regimen C: 2 weeks of AmB plus 5-FC, Regimen D: 2 weeks of AmB plus fluconazole), given the vast differences between resource-limited and resource-abundant settings regarding therapeutic drug accessibility, availability, and affordability for HIV-associated (CM).

**Methods:**

We conducted a network meta-analysis to compare the therapeutic efficacy and safety of four different induction treatment regimens.

**Results:**

The 10-week mortality of Regimen A was significantly lower than that of Regimen B and D, and the 2-week mortality of Regimen A was significantly lower than that of Regimen B. Furthermore, there were no statistically significant differences in 10-week mortality, 2-week mortality, as well as in effective fungicidal activity (EFA) over the first 2 weeks among Regimens B, C, and D. The statistical differences in adverse events between Regimen B and Regimen D, and Regimen C and Regimen D were not calculated to be significant.

**Conclusions:**

Our results indicate that, 1 week of AmB plus 5-FC followed by 1 week of fluconazole is superior to the three other studied regimens, and that when 5-FC is not available, accessible, or affordable, 2 weeks of AmB plus fluconazole or 1 week of AmB plus fluconazole followed by 1 week of fluconazole is an appropriate substitution for 2 weeks of AmB plus 5-FC.

## Introduction

Cryptococcal meningitis (CM) remains a significant contributor to human immunodeficiency virus- (HIV-) associated mortality (Lawrence et al., 2019), accounting for 15% mortality overall globally (Rajasingham et al., 2017). In 2014, an estimated 223,100 HIV-associated CM cases and 181,100 deaths occurred globally (Lawrence et al., 2019), 163,000 of these HIV-associated CM (73%) patients were diagnosed in sub-Saharan Africa and Southeast Asia (Oladele et al., 2017; Rajasingham et al., 2017).

Prompt, rational, and effective antifungal treatment is imperative to HIV-associated CM management (WHO, 2018). HIV-associated CM treatment can be divided into an induction phase, a consolidation phase, and a maintenance phase (Rhein et al., 2019). The object of the induction phase is to drastically reduce cerebrospinal fluid (CSF) fungal burden, and is critical for patients’ survival (Boyer-Chammard et al., 2019). The WHO (2018) guidelines for the preferred induction treatment regimens for HIV-associated CM was updated in March 2018, changing Amphotericin (AmB) plus flucytosine (5-FC) for 2 weeks or AmB plus fluconazole for 2 weeks, to 1 week of AmB plus 5-FC, followed by 1 week of fluconazole. The reason for this change was to reduce the potential for therapeutic drug toxicity, and to reduce the cost of treatment while maintaining efficacy (Tenforde et al., 2018).

The WHO guidelines recommendation change was mainly based on the evidence of a network meta-analysis evaluating the best regimen for patients with HIV-associated CM (Tenforde et al., 2018). This meta-analysis found that 1 week of AmB plus 5-FC, followed by fluconazole for a week was probably superior to other regimens. However, early phase study (Jarvis et al., 2019) of novel liposomal AmB (as opposed to AmB deoxycholate) at many African centers will determine the relative efficacy of this new regimen, but does not offer any new information beyond the network meta-analysis, and some results will be included in our study for analysis. In addition, 5-FC is neither readily accessible nor affordable in resource-limited settings, where a typically heavy burden of HIV-associated CM prevails. Another network meta-analysis (Yao et al., 2014) investigating the efficacy of 5-FC plus AmB and fluconazole plus AmB, demonstrated no difference in mortality between the two regimens at 3 months of treatment. These studies indicate that the most appropriate, effective, and readily available induction regimen for HIV-associated CM in resource-limited settings has, as yet, not been fully addressed, and therefore warrants further investigation.

In the present network meta-analysis, we compared three WHO recommended regimens (Rapid Advice: Diagnosis, Prevention and Management of Cryptococcal Disease in HIV-Infected Adults, Adolescents and Children.SourceGeneva: World Health Organization, 2011; WHO, 2018), and a commonly chosen 5-FC-free regimen in clinical practice, with the objective of finding the most appropriate antifungal regimen for the induction treatment of HIV-associated CM. The regimens we chose to compare included 1 week of AmB plus 5-FC followed by 1 week of fluconazole (Regimen A), 1 week of AmB plus fluconazole followed by 1 week of fluconazole (Regimen B), 2 weeks of AmB plus 5-FC (Regimen C), and 2 weeks of AmB plus fluconazole (Regimen D).

## Method

### Search Strategy

Randomized controlled trials (RCTs) or cohort studies recruiting HIV-associated CM patients were searched and screened in Pubmed, the Cochrane Library, Web of Science, and EBSCOhost/MEDLINE from inception until Nov 15^th^, 2019. Keywords connected as “HIV OR AIDS OR human immunodeficiency virus AND cryptococcal meningitis AND (RCT or cohort study)” were used for search in titles and abstracts (Bicanic et al., 2009; Moher et al., 2015).

### Inclusion Criteria

RCTs or cohort studies were included if they satisfied the following criteria:

1. Study participants were HIV-positive patients with their first episode of CM, and CM was diagnosed by isolation of cryptococcus from CSF cultures, positive CSF India ink staining, and/or positive CSF cryptococcal antigen tests, or isolation of cryptococcus in blood culture with clinical presentations of meningo-encephalitis and typical CSF features (Zheng et al., 2015; Kanters et al., 2016; Spec and Powderly, 2018).
2. Patients receiving 1 week of AmB plus 5-FC followed by 1 week of fluconazole, 1 week of AmB plus fluconazole followed by 1 week of fluconazole, 2 weeks of AmB plus 5-FC, and 2 weeks of AmB plus fluconazole were included.
3. Studies reported 10-week mortality and one of the following outcomes: 2-week mortality, effective fungicidal activity (EFA) over the first 2 weeks, adverse events, and drug cost.

### Exclusion Criteria

Studies were excluded if:

1. They were not RCTs or cohort studies.
2. Study participants were HIV-negative.
3. The diagnosis of CM was not clearly established by positive CSF cultures, positive India ink staining, or positive blood cultures of cryptococcus in association with clinical presentation and CSF features of CM.
4. The study regimens were for consolidation treatment, maintenance treatment, pre-emptive antifungal treatment, or secondary prophylaxis, in the absence of data for induction treatment.
5. Cases were those of recurrence or retreatment, and not those of first episode of CM.
6. They were not published in English.

### Data Extraction

We extracted the following data for further analysis: (1) characteristics of studies: first author, date of publication, study type, study duration, and country/area; (2) characteristics of patients: number, age, timing of ART initiation after antifungal therapy; (3) interventions included induction therapeutic regimens and consolidation therapeutic regimens; (4) outcomes included 10-week mortality, 2-week mortality, EFA over the first 2 weeks (Pullen et al., 2020), adverse events, and drug cost. Induction therapeutic regimens were as follows: 1 week of AmB plus 5-FC followed by 1 week of fluconazole (Regimen A), 1 week of AmB plus fluconazole followed by 1 week of fluconazole (Regimen B), 2 weeks of AmB plus 5-FC (Regimen C), and 2 weeks of AmB plus fluconazole (Regimen D).

### Study Quality Assessment

Two review authors (YL and YQ) independently evaluated the methodological quality of the six RCTs included in our network meta-analysis by means of the Cochrane “risk of bias” tool (Higgins et al., 2011; Huang et al., 2018). The following seven measures of bias were assessed at “low risk,” “unclear risk,” or “high risk”: random sequence generation (selection bias), allocation concealment (selection bias), blinding of participants and researchers (performance bias), blinding of outcome assessment (detection bias), incomplete outcome data (attrition bias), selective reporting (reporting bias), and other bias.

### Measurement of Treatment Effect

Ten-week mortality and 2-week mortality for Regimens A, B, C, and D was assessed using forest plots. Risk ratios (RRs) with 95% confidence intervals (CIs) in each pair comparison were generated for treatment effect measurements (Bicanic et al., 2015). Adverse events were also assessed using forest plots and risk ratio (RRs) with 95% Cis (Beardsley et al., 2016; Rhein et al., 2016). EFA over the first 2 weeks was assessed by mean differences (MDs) with 95% Cis (Loyse et al., 2012; Tenforde et al., 2018).

### Relative Ranking of Network Meta-Analysis

The cumulative ranking probabilities were summarized using the surface under the cumulative ranking area (SUCRA) curve (Salanti et al., 2011; Chaimani et al., 2013). The SUCRA value represents the surface under the cumulative ranking curve, and the probability for each regimen to be the best option for HIV-associated CM. The larger the SUCRA value, the higher the ranking probability for the regimen in the network (Tenforde et al., 2018).

### Assessment of Consistency and Heterogeneity

Consistency of network meta-analysis was assessed by global and local consistency (Cipriani et al., 2009). Global consistency was assessed by the consistency model and inconsistency model (Tonin et al., 2017). Local consistency was assessed by the node-splitting method (White, 2015).

Statistical heterogeneity was assessed using the inconsistency factor (IF) and *p*-values in loop-specific heterogeneity (Shim et al., 2017). Factors such as dosing within each regimen in the induction and consolidation therapeutic phase, ART status, and risk of bias (assessed by “risk of bias” tool) were considered likely to cause clinical and methodological heterogeneity (Tenforde et al., 2018).

Reporting bias was assessed by examining asymmetry in funnel plots of pairwise comparisons (Tenforde et al., 2018).

### Data Analysis and Synthesis

Pairwise meta-analysis was conducted with the traditional frequentist approach (Tu and Wu, 2017). The command “networkplot” was used for pairwise meta-analysis by STATA Version 15.1 (Statacorp, Texas, USA) (StataCorp, 2017; White, 2015).

For dichotomous outcome measures, RRs were calculated with 95% CIs. For continuous outcomes, MDs were calculated with 95% CIs. These two types of outcomes were both analyzed by Review Manager Version 5.3 (The Nordic Cochrane Centre, The Cochrane Collaboration, Copenhagen) (Copenhagen: The Nordic Cochrane Centre, The Cochrane Collaboration, 2014).

Preferred Reporting Items for Systematic Reviews and Meta-Analyses (PRISMA) guidelines were followed through all phases in our study (Bicanic et al., 2009; WHO, 2018). Registration number CRD42019124942 was obtained after registering the protocol in the PROSPERO international prospective register of systematic reviews (PROSPERO International prospective register of systematic reviews).

### Results

**Figure 1:**
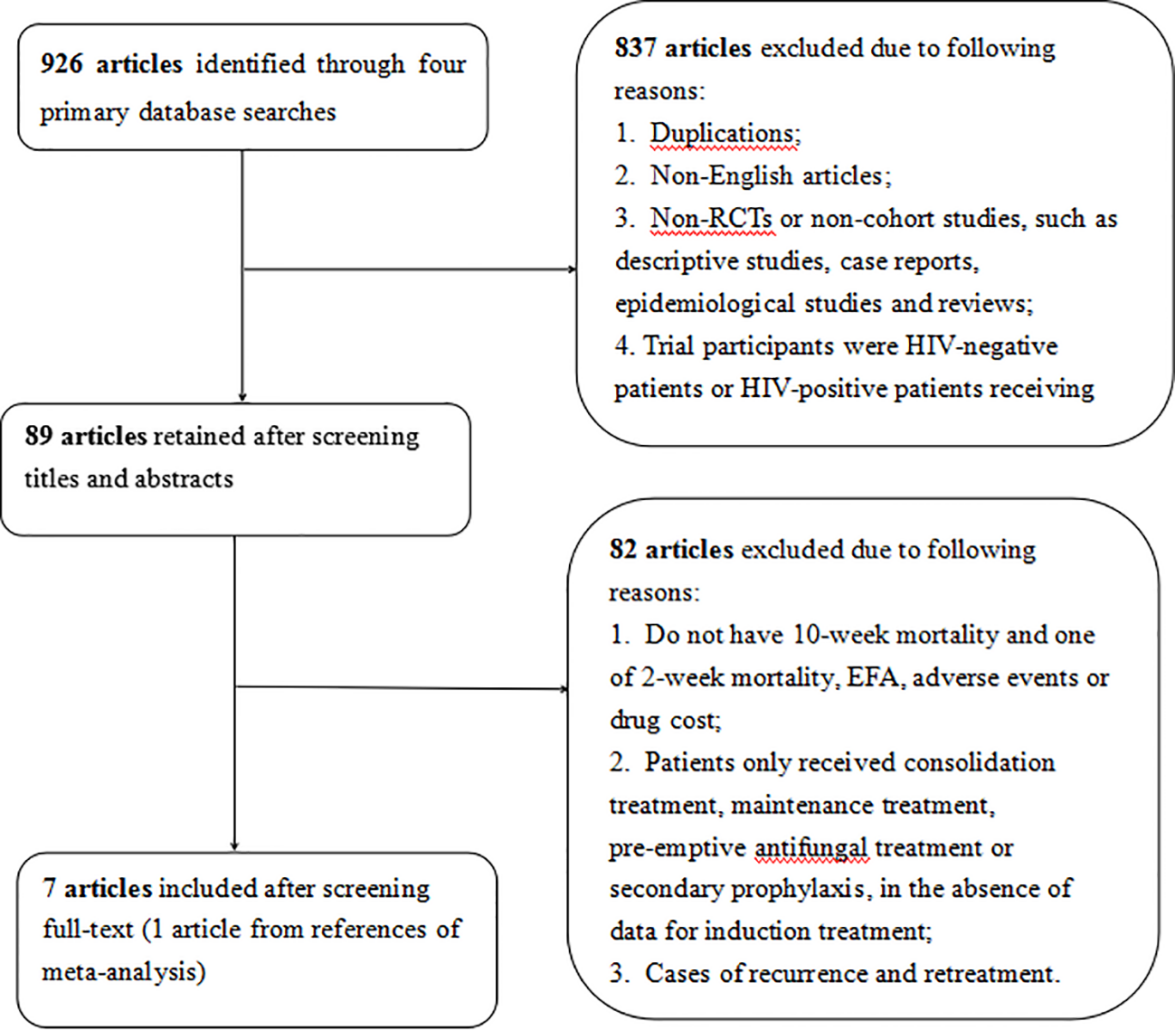
Flow chart of study selection.

A total of 926 articles were retrieved from four electronic databases through our search strategy, and 89 of these articles were eligible for inclusion after screening titles and abstracts. Among the 89 articles, 82 were excluded due to not reporting 10-week mortality, or one of the following criteria: 2-week mortality, EFA, adverse events and drug cost, absence of data for induction treatment, or recurrence and retreatment. Finally, seven articles (Brouwer et al., 2004; Tansuphaswadikul et al., 2006; Loyse et al., 2012; Rajasingham et al., 2012; Day et al., 2013; Molloy et al., 2018; Jarvis et al., 2019) were included into our network meta-analysis after full-text reviewing (**Figure 1**). In the seven incorporated studies, one arm received 1 week of AmB plus 5-FC followed by 1 week of fluconazole, four arms received 1 week of AmB plus fluconazole followed by 1 week of fluconazole, five arms received 2 weeks of AmB plus 5-FC, and seven arms received 2 weeks of AmB plus fluconazole (**Table 1**).

**Table 1:** Characteristics of six included studies.

Study (author year)	Number	Study type	Study duration	Country/area	Age (years old)	Induction therapeutic regimens	Consolidation therapeutic regimens	ART initial time (after antifungal therapy)	2-week mortality	10-week mortality	EFA over the first 2 weeks (log CFU/ml/day)	Adverse events (AEs) over the first 2 weeks	Drug cost
(Molloy et al., 2018)	453	a RCT	From 2013/01 to 2016/11	9 African centers	≥18	(1) AmB (1 mg/kg/day, 1 week) + 5-FC (100 mg/kg/d), 2 weeks; (2) AmB (1 mg/kg/day, 1 week) + fluconazole (1,200 mg/d), 2 weeks; (3) AmB (1 mg/kg/day) + 5-FC (100 mg/kg/d), 2 weeks; (4) AmB (1 mg/kg/day) + fluconazole (1,200 mg/d), 2 weeks	Fluconazole (800 mg/d until ART initiation at 4 weeks, at 400 mg/d until 10 weeks)	At 4 weeks	(1) 36 out of 111; (2) 13 out of 113; (3) 25 out of 114; (4) 24 out of 115	(1) 54 out of 111; (2) 27 out of 113; (3) 47 out of 114; (4) 44 out of 115	(1) −0.41 ± 0.26; (2) −0.39 ± 0.22; (3) −0.49 ± 0.26; (4) −0.37 ± 0.24	128 grade 3 or 4 AEs occurred in group (1) and (2); 154 grade 3 or 4 AEs occurred in group (3) and (4)	Not report
(Jarvis et al., 2019)^b^	79	RCT	From 2014/10 to 2016/9	Botswana and Tanzania	≥18	(1) L-AmB (10 mg/kg, on day 1) + fluconazole (1,200 mg/d), 2 weeks; (2) L-AmB (10 mg/kg, on day 1 and 3) + fluconazole (1,200 mg/d), 2 weeks; (3) L-AmB (10 mg/kg, on day 1, 3 and 7) + fluconazole (1,200 mg/d), 2 weeks; (4) L-AmB (3 mg/kg/day) + fluconazole (1,200 mg/d), 2 weeks	fluconazole (800 mg/day until 10 weeks)	ART naive and exposed	(1) 2 out of 18; (2) 3 out of 20; (3) 5 out of 20; (4) 2 out of 21	(1) 4 out of 18; (2) 3 out of 20; (3) 10 out of 20; (4) 6 out of 21	(1) −0.52 ± 0.35; (2) −0.47 ± 0.29; (3) −0.54 ± 0.44; (4) −0.41 ± 0.11	(1) 2 grade 3 or 4 AEs out of 18; (2) 3 grade 3 or 4 AEs out of 20; (3) 4 grade 3 or 4 AEs out of 20; (4) 4 grade 3 or 4 AEs out of 21	Not report
(Tansuphaswadikul et al., 2006)	57	RCT	From 2002/11 to 2003/9	Thailand	>13	(1) AmB (0.7 mg/kg/day, 1 week) + fluconazole (400 mg/d), for 2 weeks; (2) AmB (0.7 mg/kg/day, 2 week) + fluconazole (400 mg/d), for 2 weeks	fluconazole (400 mg/day until at least one CSF culture shows negative or until 8 weeks)	On ART	Not reported	(1) 2 out of 30; (2) 6 out of 27	Not reported	All regimens were well tolerated and no drawn within the first two weeks because of side effects.	Not reported
(Rajasingham et al., 2012)	561	System-atic review	No reported	South Africa, Uganda, and Thailand	No specified	(1) AmB (1 mg/kg/d, 5–7 days) + fluconazole (1,200 mg/d), for 2 weeks; (2) AmB (0.7–1 mg/kg/d) + fluconazole (800 mg/d), for 2 weeks; (3) AmB (0.7–1 mg/kg/d) + 5-FC (100 mg/kg/d), for 2 weeks	Not reported	On ART	Not reported	(1) 33 out of 127; (2) 61 out of 203; (3) 62 out of 231	Not reported	Not reported	Total cost of care: (a) $217.58 (b) $402.07 (c) $467.48
(Loyse et al., 2012)^c^	67	RCT	From 2006/08 to 2008/10	South Africa	≥18	(1) AmB (0.7–1 mg/kg) + 5-FC (100 mg/kg/d), for 2 weeks; (2) AmB (0.7–1 mg/kg) + fluconazole (800 mg/d), for 2 weeks; (3) AmB (0.7–1 mg/kg) + fluconazole (600 mg twice daily), for 2 weeks.	fluconazole (400 mg/d for 8 weeks and 200 mg/d thereafter)	2 weeks	(1) 1 out of 20; (2) 7 out of 45	(1) 6 out of 20; (2) 13 out of 45	(1) -0.41 ± 0.22; (2) -0.39 ± 0.26	(1) 9 grade 3 or 4 AEs in group 1; (2) 14 grade 3 or 4 AEs in group 2; (3) 10 grade 3 or 4 AEs in group 3	Not reported
(Brouwer et al., 2004)	32	RCT	From 2002/05 to 2002/12	Thailand	No	(1) AmB (0.7 mg/kg/d) + 5-FC (100 mg/kg/d), for 2 weeks; (2) AmB (0.7 mg/kg/d) + fluconazole (400 mg/d), for 2 weeks;	fluconazole (400 mg/d for 8 weeks, and 200 mg/d thereafter)	After 10 weeks	(1) 1 in 16; (2) 5 in 16;	(1) 1 in 16; (2) 7 in 16	(1) −0.54 ± 0.19; (2) −0.39 ± 0.15	No withdrawn within the first 2 weeks because of side-effects	Not reported
(Day et al., 2013)	199	RCT	From 2004/4 to 2010/09	Vietnam	>14	(1) AmB (1 mg/kg) + 5-FC (100 mg/kg/d), for 2 weeks; (2) AmB (1 mg/kg) + fluconazole (400 mg twice daily), for 2 weeks	(1) fluconazole (400 m/d for 8 weeks); (2) fluconazole (400 mg/d for 8 weeks)	Some at study entry; some within 2 weeks; some between 2 to 10 weeks	(1) 15 out of 100; (2) 20 out of 99	(1) 30 out of 100; (2) 33 out of 99	(1) −0.42 ± 0.20; (2) −0.32 ± 0.25	85 adverse events both in group (1) and group (2)	Not reported

^a^RCT, randomized controlled trial.

^b^Arm (1), (2), and (3), which have same dose of L-AmB, and different course (single, 2, or 3 dose) of L-AmB but all less than 1 week, were combined as Regimen B in Jarvis et al.’s study. The mean EFA of arm (1), (2), and (3) was calculated as the EFA of Regimen A in Jarvis et al.’s study.

^c^Arm (2) and arm (3), which have the same course and same dose of AmB and same course but different dose of fluconazole (800 mg/d vs. 600 mg/d), were combined as Regimen D in Loyse et al.’s study. The mean EFA of arm (2) and arm (3) was calculated as the EFA of Regimen D in Loyse et al.’s study.

The risk bias of 6 RCTs described as “low risk,” “high risk,” or “unclear risk” with supporting evidence is presented in **Supplementary Material Table**. The results showed that the high risk of performance bias (non-blinding of participants and researchers) of six included RCTs may have contributed to the risk of bias.

### Assessment of Ten-Week and Two-Week Mortality

**Figure 2:**
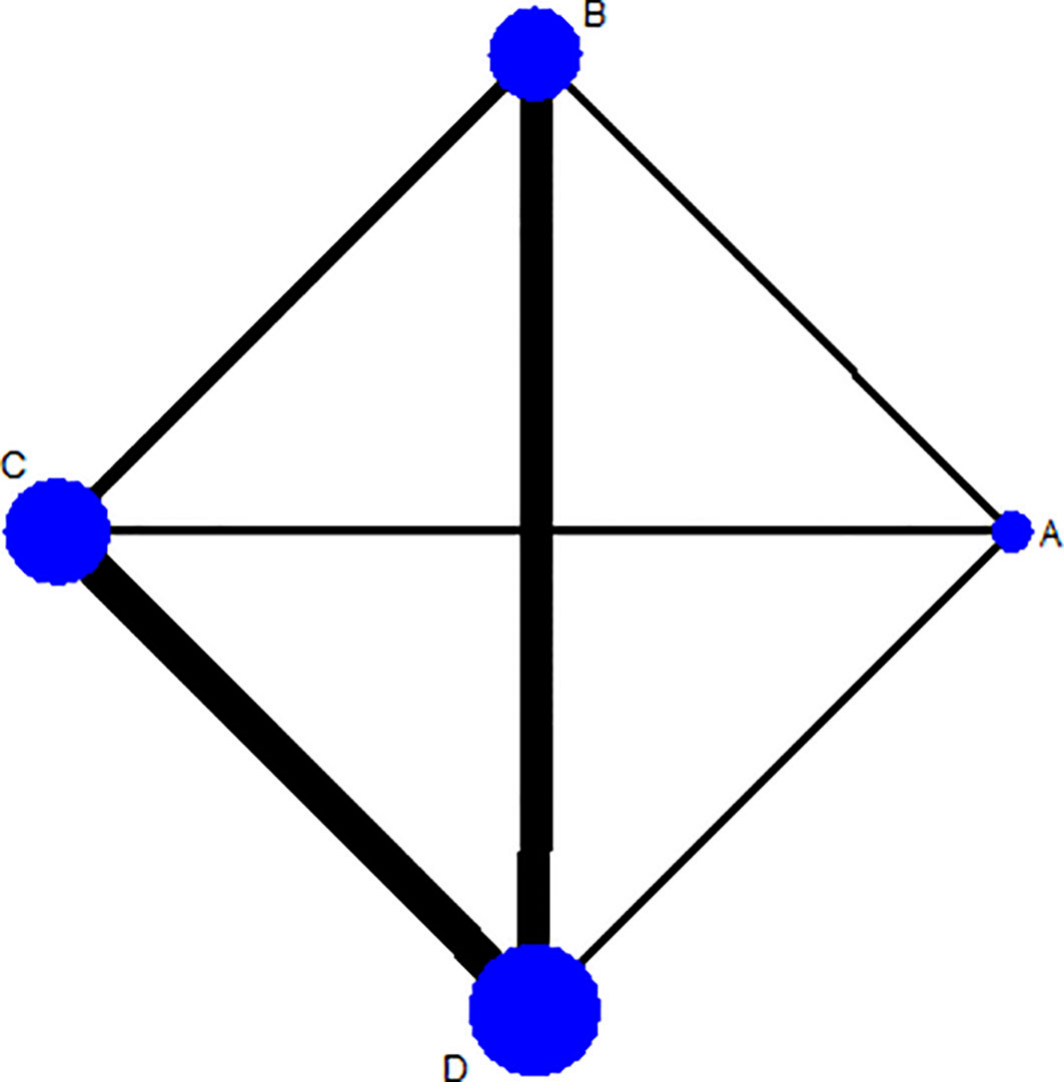
Network plot for 10-week mortality among Regimens A, B, C, and D. Regimen A: 1 week of AmB plus 5-FC followed by 1 week of fluconazole; Regimen B: 1 week of AmB plus fluconazole followed by 1 week of fluconazole; Regimen C: 2 weeks of AmB plus 5-FC; Regimen D: 2 weeks of AmB plus fluconazole. Note: The thicker the line, the more studies of the comparison between 2 interventions.

Ten-week mortality of seven trials with six pairwise comparisons (1,448 persons) was assessed by STATA 15.1, forming a rhombus-shaped network (**Figure 2**). An evidence contribution graph denoting the degree of influence of direct comparisons on the whole network is shown in **Supplementary Material Figure 1**. This suggested that comparison C *vs.* D contributed most to the entire network (66.0%), followed by comparison B *vs.* D (35.3%) and comparison A *vs.* B (30.7%).

**Figure 3:**
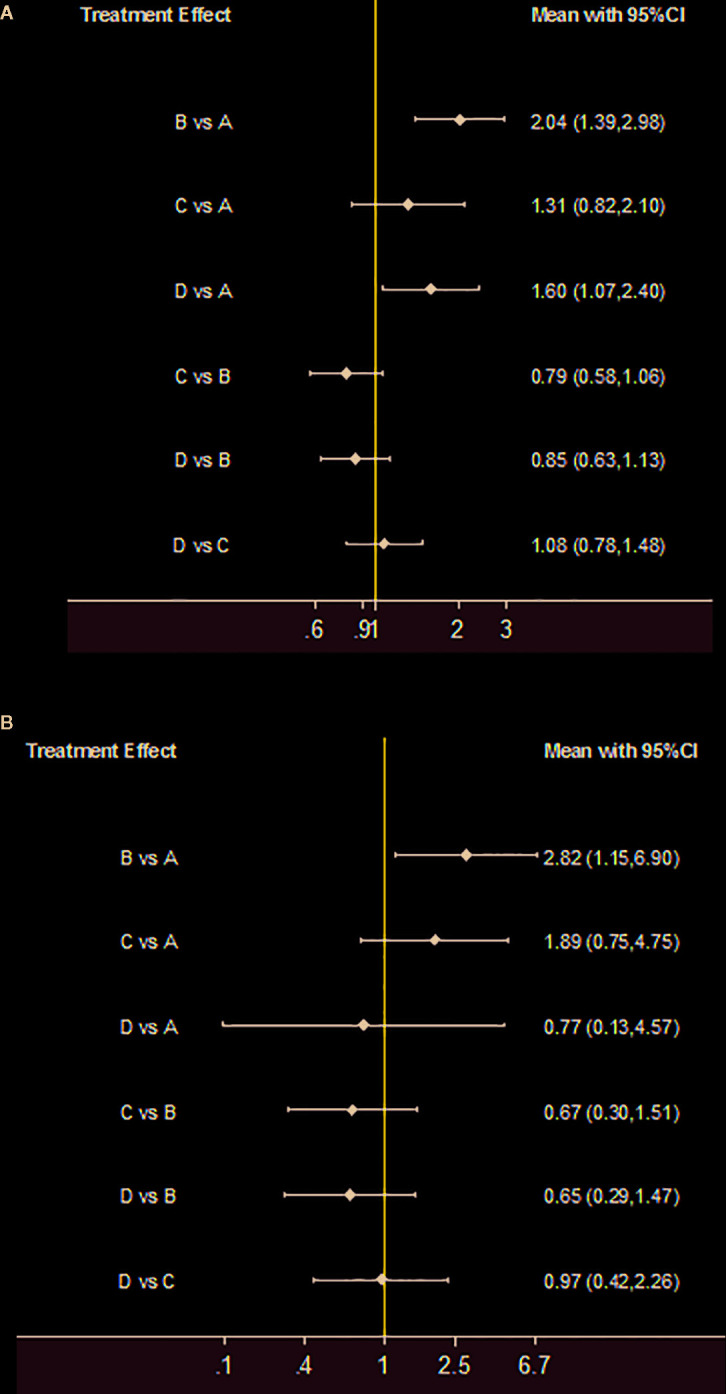
Treatment effect. **(A)** 10-week mortality for each comparison; **(B)** 2-week mortality for each comparison. If the risk ratio with 95%CI of a comparison is greater than 1, indicating the results of comparison favored the Regimen.

The 10-week mortality of the six pairwise comparisons was displayed in a forest plot (**Figure 3A**). The RRs of Regimen A *vs*. Regimen B, and Regimen A *vs.* Regimen D with 95% CIs were 2.04 (1.39, 2.98) and 1.60 (1.07, 2.40), all greater than 1, indicating the 10-week mortality of each of the comparisons all favored Regimen A. Meanwhile, the calculated differences in 10-week mortality between regimens A and B, and regimens A and D, were both statistically significant. However, the wide enough CIs of comparisons A *vs.* C, B *vs.* C, B *vs.* D, and C *vs.* D crossed the line of no effect (**Figure 3A**), suggesting that the differences in 10-week mortality between these specific pairwise comparisons were all not significant.

Two-week mortality of four trials was compared and presented in forest plots (**Figure 3B**). The 2-week mortality of Regimen A had a noticeable advantage when compared with Regimen B (2.82, 95% CI 1.15 to 6.90). There were no significant statistical differences in 2-week mortality among Regimens B, C, and D.

### Relative Ranking of 10-Week Mortality

**Figure 4:**
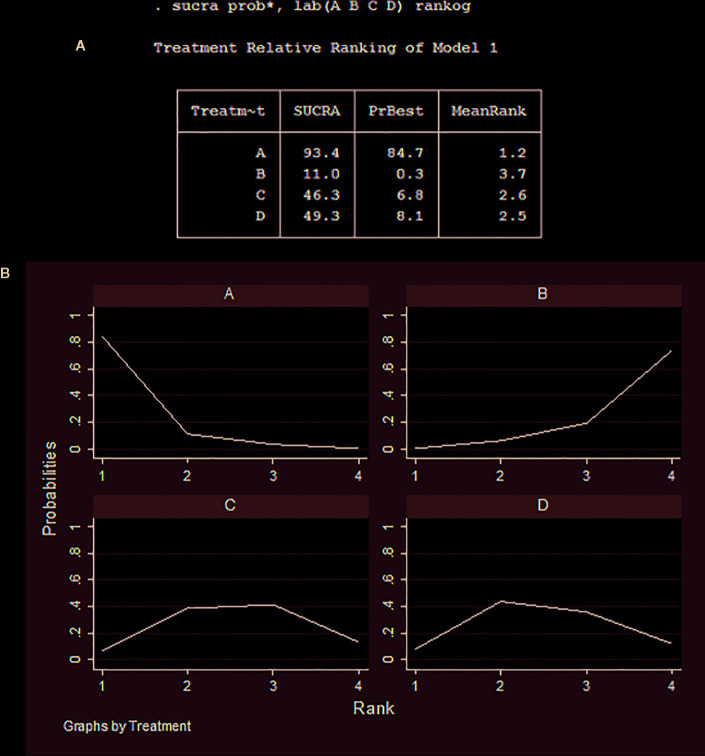
Cumulative ranking probability of 10-week mortality for each regimen. **(A)** Relative ranking of regimens A, B, C, and D; **(B)** The surface under the cumulative ranking area of regimens A, B, C, and D.

Cumulative ranking probabilities of 10-week mortality for each treatment regimen are shown in **Figures 4A, B**. The SUCRA values for each regimen were as follows: Regimen A (93.4%), Regimen B (11.0%), Regimen C (46.3%), and Regimen D (49.3%). Thus, the optimal induction treatment regimen options for HIV-associated CM, ranked in the order of largest to smallest SUCRA probabilities, are A>D>C>B.

### Treatment Effect for EFA Over the First 2 Weeks Among Regimens B, C, and D

**Figure 5:**
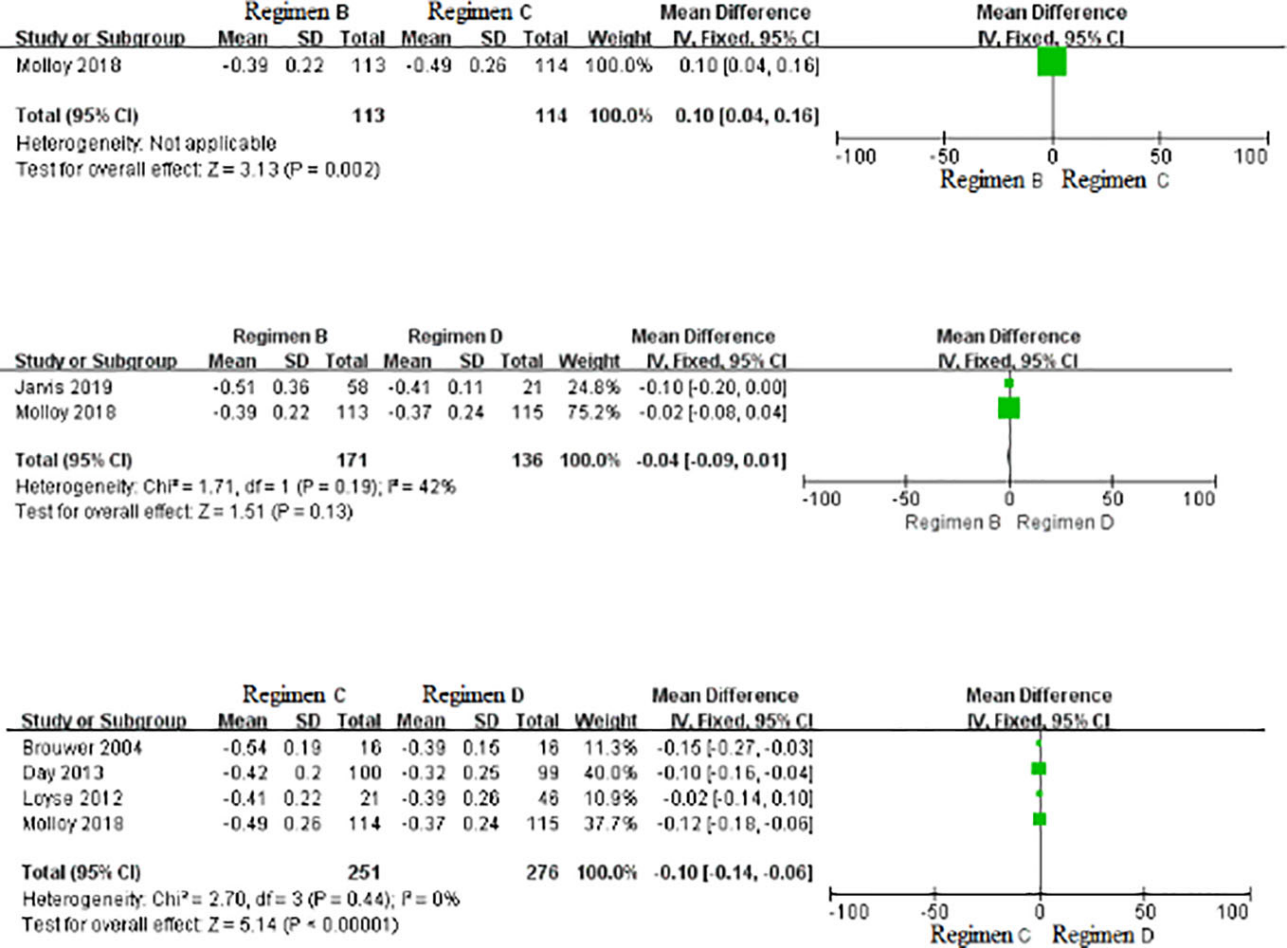
Forest plot of comparisons of EFA over the first 2 weeks. SD, standard deviation.

As displayed in **Figure 5**, no statistically significant differences were found in EFA among Regimens B, C, and D.

### Adverse Events

**Figure 6:**
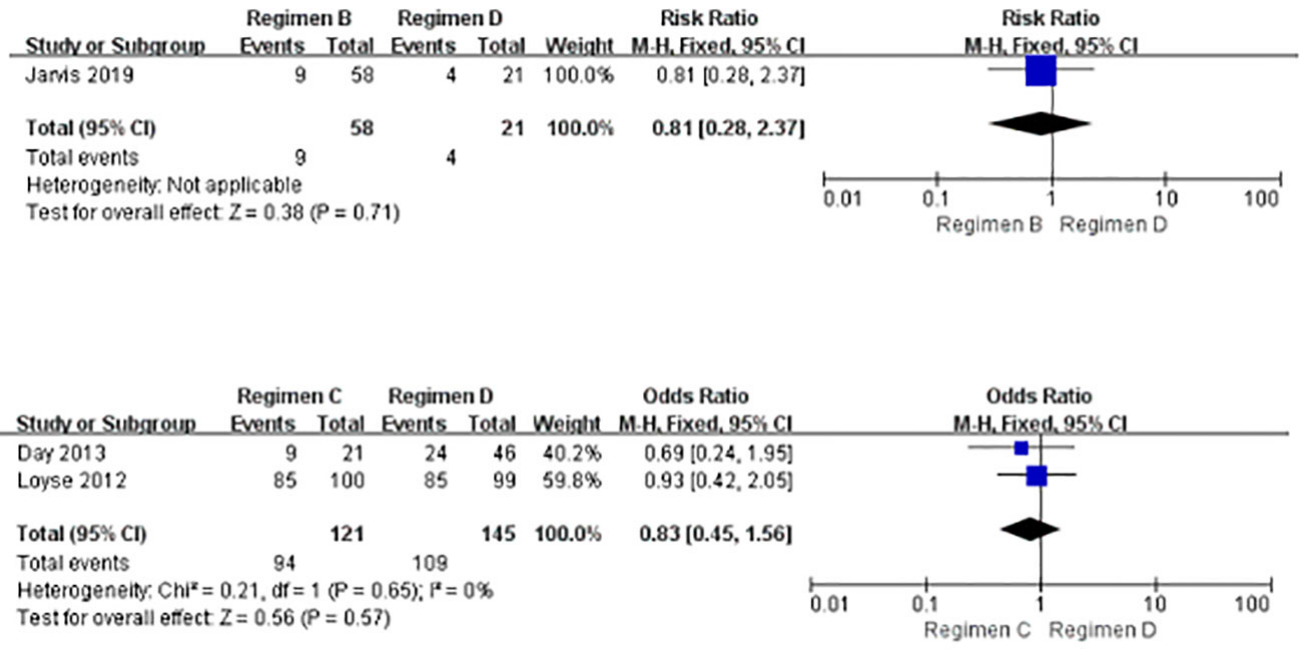
Forest plot of comparisons of adverse events.

As displayed in **Figure 6**, there were no observable statistical differences in adverse events between Regimen B and Regimen D, and between Regimen C and Regimen D. None of the included studies reported comparisons of adverse events between regimens B and C.

### Consistency and Heterogeneity of Network Meta-Analysis

Consistency was assessed using 10-week mortality data, and was shown in **Supplementary Material Figures 2A–C**. The result of global consistency, which was conducted *via* a consistency model, indicated that inconsistency was not significant (*p* = 2.56, >0.05) (**Supplementary Material Figure 2A**). Thus, the consistency model could be used for further analysis.

Loop-specific heterogeneity was evaluated by calculating IF *via* the Lumley method. The closer the IF value is to 0, the better the consistency is between direct and indirect evidence (**Supplementary Material Figure 3**). In our results, the IF of loop A-C-D was 0.00, and that of loop B-C-D was 0.07, indicating negligible inconsistency between direct and indirect evidence of these two loops. On the other hand, the *p-* values for loop A-B-C and loop A-B-D were 0.37 and 0.33 (both >0.05) respectively (**Supplementary Material Figure 3**), indicating that low inconsistency exists between direct and indirect comparisons of these loops (Tenforde et al., 2018).

### Bias of Network Meta-Analysis

The reporting bias funnel plot of six comparisons is displayed in **Supplementary Material Figure 4**. The results showed that most scatter points were located above the funnel plot, and were evenly distributed on both sides of the red indicator line, suggesting that reporting bias was negligible. However, the high risk of detection bias (non-blinding of outcome assessment) and attrition bias (incomplete outcome data) of six included RCTs may have caused some bias to our results (**Supplementary Material Table 1**).

## Discussion

In this study, we conducted a network meta-analysis to compare the appropriateness of three WHO-recommended induction therapeutic regimens (regimens A, C, and D), and a 5-FC-free induction therapeutic regimen (regimen B) usually chosen in clinical practice in resource-limited settings. All trials included in our study were carried out in resource-limited settings, and therefore, our results would be of clinical significance for the treatment of HIV-associated CM in those specific settings.

The 10-week mortality of Regimen A was significantly lower than that of Regimen B and Regimen D, and the 2-week mortality of Regimen A was significantly lower than that of Regimen B. The above results are consistent with the results of another network meta-analysis conducted by Tenforde et al. (2018), even though the included trials in the Tenforde et al. network meta-analysis, as well as those included in our analysis, were inconsistent. In addition, our relative ranking results showed that the probability for Regimen A to be the most appropriate option amongst the four studied options for the treatment of HIV-associated CM was 93.4%, which reinforces our confidence in the utilization of this latest regimen recommendation by the WHO in resource-limited settings. As for 10-week mortality, consolidation therapy also plays an important role other than induction therapy. In our study, patients in the included six studies were all treated with fluconazole for consolidation therapy. The administered doses ranged from 400 to 800 mg/day, and the consolidation course duration ranged from 8 to 10 weeks.

Our results of drug effectiveness suggest that 5-FC as a second drug is superior to fluconazole when combined with AmB, because Regimen A (1 week of AmB plus 5-FC followed by 1 week of fluconazole) was found to be more efficacious than Regimen B (1 week of AmB plus fluconazole followed by 1 week of fluconazole). Unfortunately, 5-FC remains neither readily accessible/available, nor affordable in resource-limited settings despite of its superior efficacy (Loyse et al., 2013a; Patel et al., 2018). Unfortunately, with widely used fluconazole monotherapy, mortality due to HIV-related CM is approximately 70% in many African low-income and middle-income countries settings (Loyse et al., 2019). In our analysis, there were no statistically significant differences in 10-week mortality and 2-week mortality among Regimen B, Regimen C, and Regimen D. There were also no statistically significant differences between these regimens in EFA over the first 2 weeks, and similarly, the differences in adverse events between Regimen B and Regimen D, and Regimen C and Regimen D were not statistically significant. Our results concur with the results of other researchers, who have observed that the antifungal combination of AmB plus fluconazole resulted in excellent yeast clearance from CSF, with few adverse events in their trials (Brouwer et al., 2004; Loyse et al., 2012), and that there were no statistically significant differences in EFA at 2 weeks of treatment between Regimen C (2 weeks of AmB plus 5-FC) and Regimen D (2 weeks of AmB plus fluconaozle) (Loyse et al., 2012). Our results, together with results of other researchers, indicate that substituting Regimen B or Regimen D for Regimen C may be a sensible choice in terms of efficacy in resource-limited settings, where 5-FC may not be available.

Furthermore, 5-FC is not licensed in 89 (71.2%) of 125 countries, and unavailable in 95 (76.0%) of 125 countries (Loyse et al., 2013b; Kneale et al., 2016). In resource-abundant settings where 5-FC is available, the cost of 5-FC varies from $4.60 to $1,409 per day (Loyse et al., 2013b). By contrast, the total treatment cost of 2 weeks of AmB plus fluconazole is only $402 per patient (Patel et al., 2018). Merry and Boulware (2016) cost-effectiveness analysis suggested 2 weeks of AmB plus fluconazole (Regimen D) was more cost-effective when compared with 2 weeks of AmB plus 5-FC (Regimen C) ($4,4605 *vs.* $75,121 per quality-adjusted life-year). Rajasingham et al.’s study (Rajasingham et al., 2012), which was based on 18 trials and cohorts in resource-limited settings, showed that 1 week of AmB with fluconazole followed by 1 week of fluconazole (Regimen B) had the best cost-effectiveness ratio, which is $20.24/quality-adjusted life years when compared with 2 weeks of AmB plus 5-FC (Regimen C) or 2 weeks of AmB plus fluconazole (Regimen D) (Rajasingham et al., 2012). The above studies have shown that even from the perspective of cost-effectiveness, substituting Regimens B or D for Regimen C remains a prudent choice in resource-limited settings.

Some limitations of our meta-analysis have to be pointed out. Firstly, one systematic review pooling the 10-week mortality and drug cost of 18 trials and cohorts was incorporated in our meta-analysis, which unfortunately had a paucity of outcome data for direct comparisons between regimens. Secondly, we did not perform an analysis of drug costs due to the limited data for medical expenditure in the included studies. Thirdly, because of the limited number of eligible studies and the limited data extracted from these studies, we did not conduct an analysis of ART use for patients in each study, nor did we evaluate the doses of antifungal medications prescribed to subjects in each study. We also did not assess the utilization of therapeutic lumbar puncture for subjects in each study, which may well have had an impact on heterogeneity in the present study. Finally, the liposomal AmB incorporated in Regimen B, and the high risk of detection bias and attrition bias may have contributed to a degree of bias to our results. Additionally, the SUCRA rankings should be interpreted cautiously, even when accompanied by statistically significant or clinically meaningful effects, due to the imprecision of treatment effects on mortality throughout the network meta-analysis (Tenforde et al., 2018).

## Conclusions and Perspectives

Regimen A (1 week of AmB plus 5-FC followed by 1 week of fluconazole) remains the most appropriate induction regimen for the treatment of HIV-associated CM in resource-limited settings, and is superior to Regimen B (1 week of AmB plus fluconazole followed by 1 week of fluconazole) and Regimen D (2 weeks of AmB plus fluconazole) in several aspects. Substituting Regimen B or Regimen D for Regimen C is appropriate in terms of efficacy in resource-limited settings, where 5-FC is likely to be unavailable or unaffordable.

## Author Contributions

YL, XY, and YC conceived and designed the protocol and study. YL and YQ identified the studies to be screened. XH identified studies for eligibility, extracted data, and assessed the methodological quality of included studies. YL performed the data analysis, with assistance from XY and YC. All authors contributed to the article and approved the submitted version.

## Funding

This work was supported by the National Science and Technology Major Project of China during the 13th Five-year Plan Period (2018ZX10302104), Key Project of Joint Medical Research Project of Science and Health in Chongqing in 2019 (2019ZDXM012).

## Conflict of Interest

All authors declare that this research was conducted in the absence of any commercial or financial relationships that could be construed as potentially causing conflict of interest.
