# Supplementary material for: What Is the Most Appropriate Induction Regimen for the Treatment of HIV-Associated Cryptococcal Meningitis When the Recommended Regimen Is Not Available? Evidence From a Network Meta-Analysis
Source: Front Pharmacol. 2020 Jun 30;11:963. doi: 10.3389/fphar.2020.00963 (PMC7344322; doi:10.3389/fphar.2020.00963)
Supplement: Supplementary file 1 [file DataSheet_1.doc]

**Supplementary material figure 1.** Contribution plot of 10-week mortality

**Supplementary material figure 2.** Global and local consistency was assessed for 10-week mortality. Global consistency was assessed by a consistency model (a) and an inconsistency model (b). Local consistency was assessed by the node-splitting method (c).

**Supplementary material figure 3.** Loop-specific heterogeneity was assessed for 10-week mortality.

**Supplementary material figure 4.** Funnel plot of reporting biases of 10-week mortality.

**Supplementary material table 1.** Risk of bias of the 5 included RCTs.

**Supplementary material figure 1.** Contribution plot of 10-week mortality

| **a** |  |
| --- | --- |
|  | **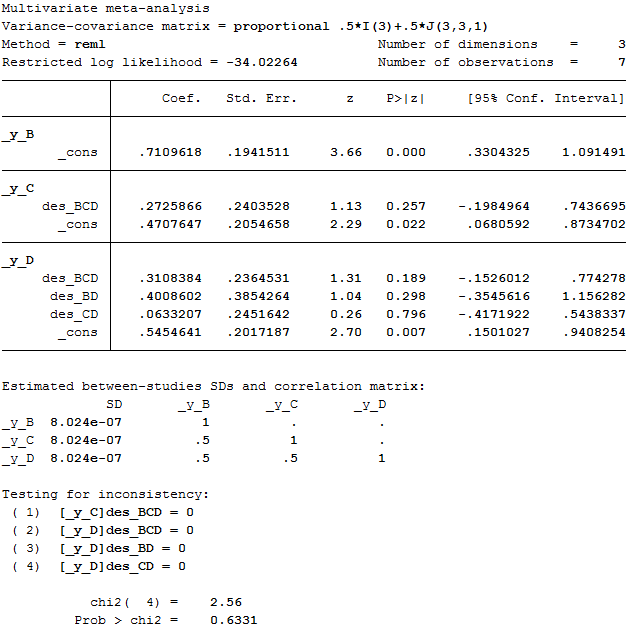** |
| **b** | **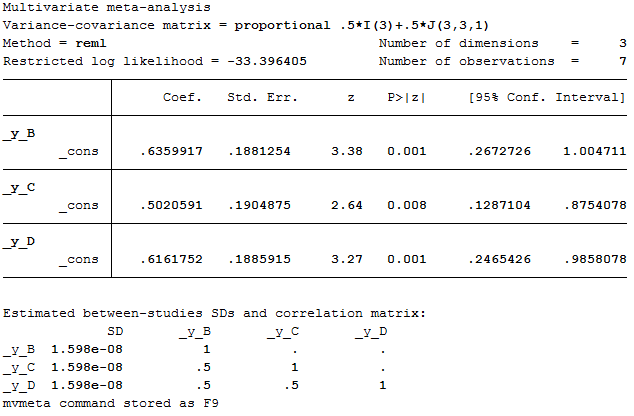** |
| **c** |  |
|  | 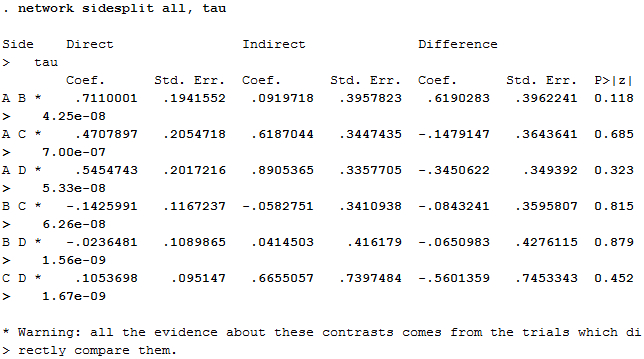 |

**Supplementary material figure 2.** Global and local consistency was assessed for 10-week mortality. Global consistency was assessed by a consistency model (a) and an inconsistency model (b). Local consistency was assessed by the node-splitting method (c).

**Supplementary material figure 3.** Loop-specific heterogeneity was assessed for 10-week mortality.

**Supplementary material figure 4.** Funnel plot of reporting biases of 10-week mortality.

**Supplementary Material table 1.** Risk of bias of the 6 included RCTs.

| **Study** | **Bias** | **Authors’ judgment** | **Supporting evidence** |
| --- | --- | --- | --- |
| Molloy 2018 | Random sequence generation (selection bias) | Low risk | “For each site, a computer-generated randomization list with block sizes of 18, 24, and 30 was produced.” |
|  | Allocation concealment (selection bias) | Low risk | For each site, a computer-generated randomization list with block sizes of18, 24, and 30 was produced. |
|  | Blinding of participants and researches (performance bias) | High risk | Open-label |
|  | Blinding of outcome assessment (detection bias) | High risk | Open-label |
|  | Incomplete outcome data (attrition bias) | Low risk | Analyses were also performed with adjustment for prespecified covariates: site, age, sex, Glasgow Coma Scale score, CD4+ cell count, CSF fungal count at baseline, and ART status at baseline. Sensitivity analyses of all-cause mortality were performed under the assumption that all the patients who were lost to follow-up had died. |
|  | Selective reporting (reporting bias) | Low risk | All prespecified outcomes were reported |
|  | Other bias | Unclear risk | No description |
| **Study** | **Bias** | **Authors’ judgment** | **Supporting evidence** |
| Jarvis 2019 | Random sequence generation (selection bias) | Low risk | Patients were block randomized individually to 1 of 4 treatment groups by means of random computer-generated lists with an allocation ratio of 1:1:1:1 and block sizes of 8. Randomization lists were created by an independent statistician who prepared sealed envelopes in advance that were sent to the sites. |
|  | Allocation concealment (selection bias) | Low risk | Patients were block randomized individually to 1 of 4 treatment groups by means of random computer-generated lists with an allocation ratio of 1:1:1:1 and block sizes of 8. Randomization lists were created by an independent statistician who prepared sealed envelopes in advance that were sent to the sites. Randomization was stratifed by abnormal mental status (Glasgow Coma Scale [GCS] score of 15 or <15) and ART status on admission at each site. |
|  | Blinding of participants and researches (performance bias) | High risk | Te patients and clinical trial team were not blinded. |
|  | Blinding of outcome assessment (detection bias) | Low risk | Laboratory staﬀ performing quantitative fungal cultures were blinded to treatment allocation. |
|  | Incomplete outcome data (attrition bias) | Low risk | EFA was calculated for 69 patients (17 in the control group, 16 in the single-dose group, 18 in the 2-dose group, and 18 in the 3-dose group). Five patients died prior to follow-up LP and 5 patients had negative baseline cultures precluding EFA calculation. 2 weeks and 10 weeks mortality and safety data were complete. |
|  | Selective reporting (reporting bias) | Low risk | EFA was calculated for 69 patients (17 in the control group, 16 in the single-dose group, 18 in the 2-dose group, and 18 in the 3-dose group). Five patients died prior to follow-up LP and 5 patients had negative baseline cultures precluding EFA calculation. 2 weeks and 10 weeks mortality and safety data were complete. |
|  | Other bias | unclear | No description |
| **Study** | **Bias** | **Authors’ judgment** | **Supporting evidence** |
| Tansuphaswadikul 2006 | Random sequence generation (selection bias) | Low risk | “The randomization was performed by a method of using the random number table list” |
|  | Allocation concealment (selection bias) | Low risk | “Study subjects were randomly allocated to either AmB1or AmB 2” |
|  | Blinding of participants and researches (performance bias) | High risk | “open-label” |
|  | Blinding of outcome assessment (detection bias) | High risk | “open-label” |
|  | Incomplete outcome data (attrition bias) | Unclear risk | “At week 10 twenty-nine patients (50.9%) had complete follow up” “Twenty patients (35.1%) were lost to follow-up, out of which 65% occurred within the first two weeks of treatment” |
|  | Selective reporting (reporting bias) | Low risk | All prespecified outcomes were reported |
|  | Other bias | Unclear risk | No description |
| **Study** | **Bias** | **Authors’ judgment** | **Supporting evidence** |
| Loyse 2012 | Random sequence generation (selection bias) | Low risk | “Randomization of patients receiving rifampicin was restricted to the first 3 arms of the study because of the significant interaction between rifampicin and voriconazole. Randomization was stratified by altered mental status at admission.” |
|  | Allocation concealment (selection bias) | Low risk | “Patients were randomized individually using a computer-generated program to 1 of 4 treatment arms” |
|  | Blinding of participants and researches (performance bias) | High risk | "Randomization was stratified by altered mental status at admission.” |
|  | Blinding of outcome assessment (detection bias) | High risk | "Randomization was stratified by altered mental status at admission.” |
|  | Incomplete outcome data (attrition bias) | Low risk | “One patient was lost to follow-up at 2–10 weeks in each of treatment arms 2, 3, and 4. One additional patient in study group 1 discontinued all medical intervention on study day 4 for personal reasons.” |
|  | Selective reporting (reporting bias) | Low risk | All prespecified outcomes were reported |
|  | Other bias | Unclear risk | No description |
| **Study** | **Bias** | **Authors’ judgment** | **Supporting evidence** |
| Brouwer 2004 | Random sequence generation (selection bias) | Low risk | “The participants were randomized in blocks of 16 by means of numbers in sealed envelopes prepared by an independent person to give equal numbers in each of four treatment arms.” |
|  | Allocation concealment (selection bias) | Low risk | “The participants were randomized in blocks of 16 by means of numbers in sealed envelopes prepared by an independent person to give equal numbers in each of four treatment arms.” |
|  | Blinding of participants and researches (performance bias) | High risk | “Treatment was not blinded” |
|  | Blinding of outcome assessment (detection bias) | High risk | “Treatment was not blinded” |
|  | Incomplete outcome data (attrition bias) | Unclear risk | No description |
|  | Selective reporting (reporting bias) | Low risk | All pre-specified outcomes were reported |
|  | Other bias | Unclear risk | No description |
| **Study** | **Bias** | **Authors’ judgment** | **Supporting evidence** |
| Day 2013 | Random sequence generation (selection bias) | Low risks | “Patients were randomly assigned to one of three induction treatments.” |
|  | Allocation concealment (selection bias) | Low risks | “A computer-generated sequence of random numbers was used to assign patients to treatment groups.” |
|  | Blinding of participants and researches (performance bias) | High risks | "Patients in group 1 received intravenous amphotericin B." |
|  | Blinding of outcome assessment (detection bias) | High risks | "Patients in group 1 received intravenous amphotericin B." |
|  | Incomplete outcome data (attrition bias) | Unclear risks | No description |
|  | Selective reporting (reporting bias) | Low risks | All pre-specified outcomes were reported |
|  | Other bias | Unclear risk | No description |
